# New G-Quadruplex-Forming Oligodeoxynucleotides Incorporating a Bifunctional Double-Ended Linker (DEL): Effects of DEL Size and ODNs Orientation on the Topology, Stability, and Molecularity of DEL-G-Quadruplexes

**DOI:** 10.3390/molecules24030654

**Published:** 2019-02-12

**Authors:** Maria Marzano, Andrea Patrizia Falanga, Stefano D’Errico, Brunella Pinto, Giovanni Nicola Roviello, Gennaro Piccialli, Giorgia Oliviero, Nicola Borbone

**Affiliations:** 1 Dipartimento di Farmacia, Università degli Studi di Napoli Federico II, via Domenico Montesano 49, 80131 Napoli, Italy; maria.marzano@unina.it (M.M.); andreapatrizia.falanga@unina.it (A.P.F.); stefano.derrico@unina.it (S.D.); picciall@unina.it (G.P.); nicola.borbone@unina.it (N.B.); 2 Dipartimento di Chimica, Università degli Studi di Milano, via Camillo Golgi 19, 20133 Milano, Italy; brunella.pinto87@gmail.com; 3 Istituto di Biostrutture e Bioimmagini, CNR, Via Tommaso De Amicis 95, 80145 Napoli, Italy; giroviel@unina.it; 4 Dipartimento di Medicina Molecolare e Biotecnologie Mediche, Università degli Studi di Napoli Federico II, via Sergio Pansini 5, 80131 Napoli, Italy

**Keywords:** G-quadruplexes, double-ended linkers, DEL-ODNs, TEL-ODNs, supramolecular G-quadruplexes, NMR, CD, size-exclusion chromatography

## Abstract

G-quadruplexes (G4s) are unusual secondary structures of DNA occurring in guanosine-rich oligodeoxynucleotide (ODN) strands that are extensively studied for their relevance to the biological processes in which they are involved. In this study, we report the synthesis of a new kind of G4-forming molecule named double-ended-linker ODN (DEL-ODN), in which two TG_4_T strands are attached to the two ends of symmetric, non-nucleotide linkers. Four DEL-ODNs differing for the incorporation of either a short or long linker and the directionality of the TG_4_T strands were synthesized, and their ability to form G4 structures and/or multimeric species was investigated by PAGE, HPLC–size-exclusion chromatography (HPLC–SEC), circular dichroism (CD), and NMR studies in comparison with the previously reported monomeric tetra-ended-linker (TEL) analogues and with the corresponding tetramolecular species (TG_4_T)_4_. The structural characterization of DEL-ODNs confirmed the formation of stable, bimolecular DEL-G4s for all DEL-ODNs, as well as of additional DEL-G4 multimers with higher molecular weights, thus suggesting a way towards the obtainment of thermally stable DNA nanostructures based on reticulated DEL-G4s.

## 1. Introduction

Among the noncanonical secondary structures adopted by nucleic acids, the G-quadruplexes (G4s) are one of the most extensively studied. G4s occur in guanosine-rich oligonucleotides (GRO) and are characterized by the presence of two or more stacked G-tetrads, planar arrangements of four guanosines held together by a cyclic array of eight Hoogsteen’s hydrogen bonds [1,2,3]. The π–π interaction generated among the stacked G-tetrads greatly stabilizes the G4s and the presence of cations, such as potassium or sodium, further contributes to the stability of G4 structures. Structural studies have demonstrated that GROs can form highly polymorphic G4 scaffolds that can differ by the number of the strands (one, two, or four) and by their mutual orientation, which lead to parallel, antiparallel, or mixed assemblies [4,5]. The wide polymorphism of G4s also arises from the length and the base composition of GROs, from the glycoside conformation of the guanosines involved in each tetrad, and from the cation species used to stabilize the complex [6,7,8]. G4 structures are involved in several relevant biological processes, such as the expression of many protooncogenes and the maintenance of telomeres length [9,10,11,12,13,14,15]. Furthermore, several aptamers, including the thrombin-binding aptamer [16,17,18,19,20] and anti-HIV-1 aptamers [21,22,23,24,25,26], adopt a G4 scaffold in their biologically active conformation. Recently, G4s emerged as interesting self-assembling scaffolds to be used in supramolecular chemistry applications and in nanotechnology for the development of new sensing probes or new materials. In addition, the G4 scaffold possesses a greater conductivity than the DNA double helix, thus suggesting its use also in bioelectronics [27,28,29,30]. It is well documented that the duplex DNA motif can be used to build supramolecular structures of various shapes and sizes by a bottom-up process named DNA origami, which is controlled by the sequence and length of the DNA strands [31,32,33]. Otherwise, supramolecular structures based on G4 building blocks are essentially confined to G4 hybrid structures, such as duplex–quadruplex repetitions and the so-called G-wires. G-wires are rod-shaped G4 superstructures in which the G4 motif can reach the length of thousands of nanometres along the axis perpendicular to the G-tetrad planes [34,35,36,37]. G-wires can be formed by the cooperative assembly of slipped G-rich ODN strands (interlocked G4s) or by the multimerization of G4 building blocks held together by end-to-end π–π stacking interactions [38,39,40,41,42,43]. 

In light of the noteworthy chemical–physical properties of the G4s, the discovery and the characterization of new supramolecular G4 assemblies represent a very interesting challenge, and the formation of the G4 scaffold and its structuring in a linear and/or reticulated topology have to be finely controlled. In fact, the main disadvantage in the design of G4-based supramolecular assemblies is the low control over the structuring and aggregation process. For these reasons, many efforts have been devoted to the design of G4-forming oligonucleotides bearing structural modifications that could allow the obtainment of new, supramolecular assemblies in a controlled fashion and that should go beyond the simple, linear rods. For example, GROs attached to the ends of branched linkers have been described, and their propensity to form monomeric or polymeric G4 structures has been investigated [44,45,46]. Several studies on branched GROs, carried out by our research group and by others, have shown that the presence of a tetra-ended linker (TEL), on which the GRO chains grow up, can positively influence the stability of the resulting G4 structures [47,48,49,50]. In particular, we demonstrated that the so-called TEL-G4s are provided with higher thermal stability and more favourable kinetic and thermodynamic parameters compared to the corresponding tetramolecular counterparts. Furthermore, we demonstrated that the TEL analogues of the G4-forming, anti-HIV aptamer having the sequence ^5′^TGGGAG^3′^ can be successfully used in place of the corresponding tetramolecular quadruplex to bind, with increased efficiency, the HIV-1 glycoprotein gp120, thus resulting in a clear enhancement of the antiviral activity of the aptamer [24,25,26].

**Scheme 1:**
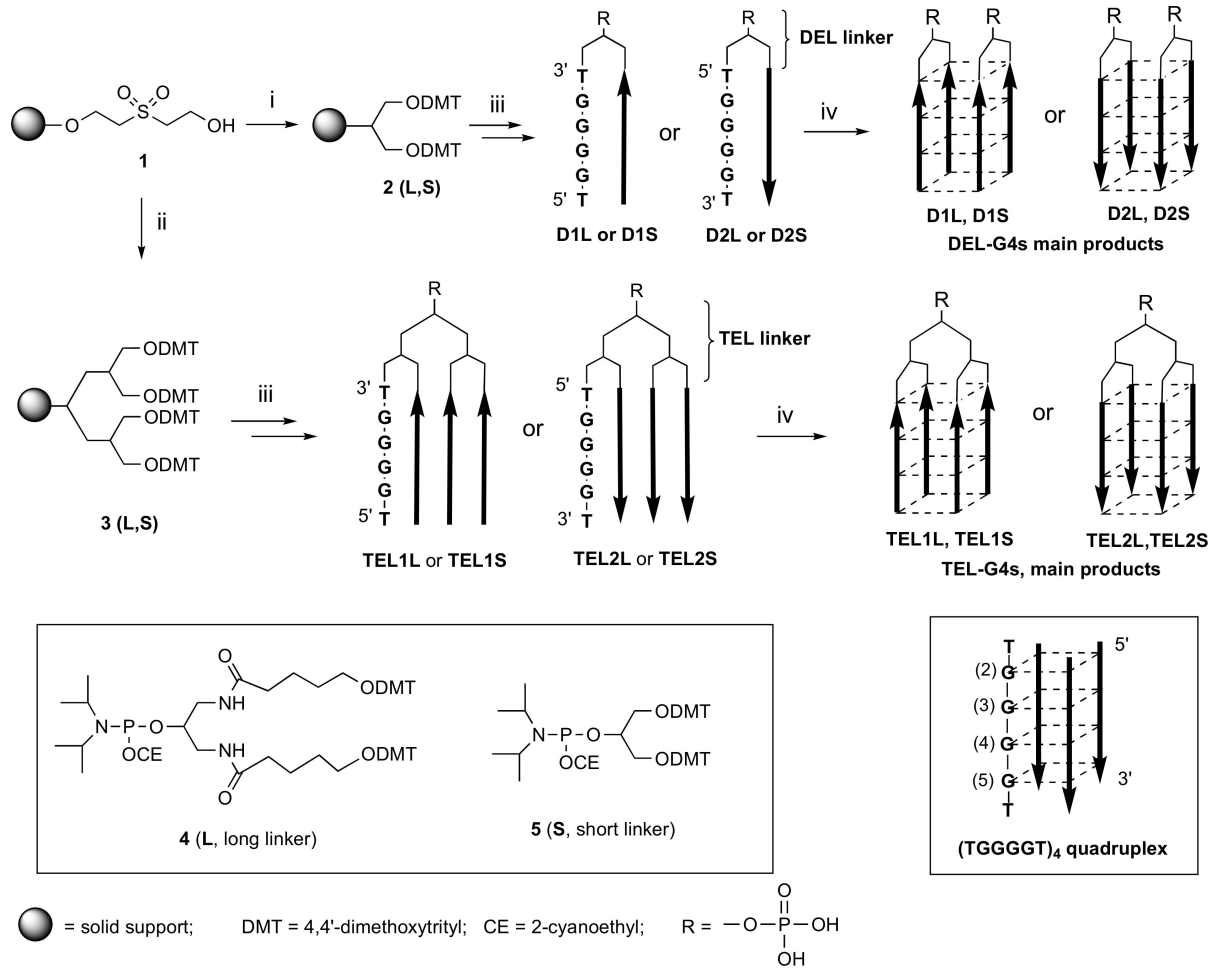
General synthetic route for compounds **D1L,S**, **D2L,S** and **TEL1L,S**, **TEL2L,S**. (i) Coupling with the phosphoramidite linker **4** or **5**; (ii) two sequential couplings with the phosphoramidite linker **4** or **5**; (iii) solid-phase DNA synthesis by phosphoramidite chemistry; iv) annealing procedure in Na^+^- or K^+^-containing buffer.

Continuing our studies on branched GROs, we report here on the synthesis and structural characterization of a new class of G4-forming oligonucleotides named double-ended-linker oligodeoxynucleotides (DEL-ODNs). The structures of DEL-ODNs, in which two TG_4_T strands are attached by either their 3′ end (**D1L,S**, Scheme 1) or 5′ end (**D2L,S)** to a symmetric, long (L) [51] or short (S) bifunctional linker, are shown in Scheme 1. As the oligonucleotide (ON) sequence, we chose TG_4_T because it forms a stable tetramolecular G-quadruplex that has been structurally characterized in detail [52,53]. Moreover, the use of the TG_4_T sequence allowed us to compare the structural properties of the here-reported DEL-G4s with those of the tetramolecular (TG_4_T)_4_ and the monomolecular TEL-(TG_4_T)_4_ (**TEL1L,S** and **TEL2L,S**, Scheme 1) corresponding G4s. The formation and properties of DEL-G4s formed by DEL-(TG_4_T)_2_ were investigated by circular dichroism (CD), native polyacrylamide gel electrophoresis (PAGE), HPLC size-exclusion chromatography (HPLC–SEC), and ^1^H-NMR experiments. 

**Figure 1:**
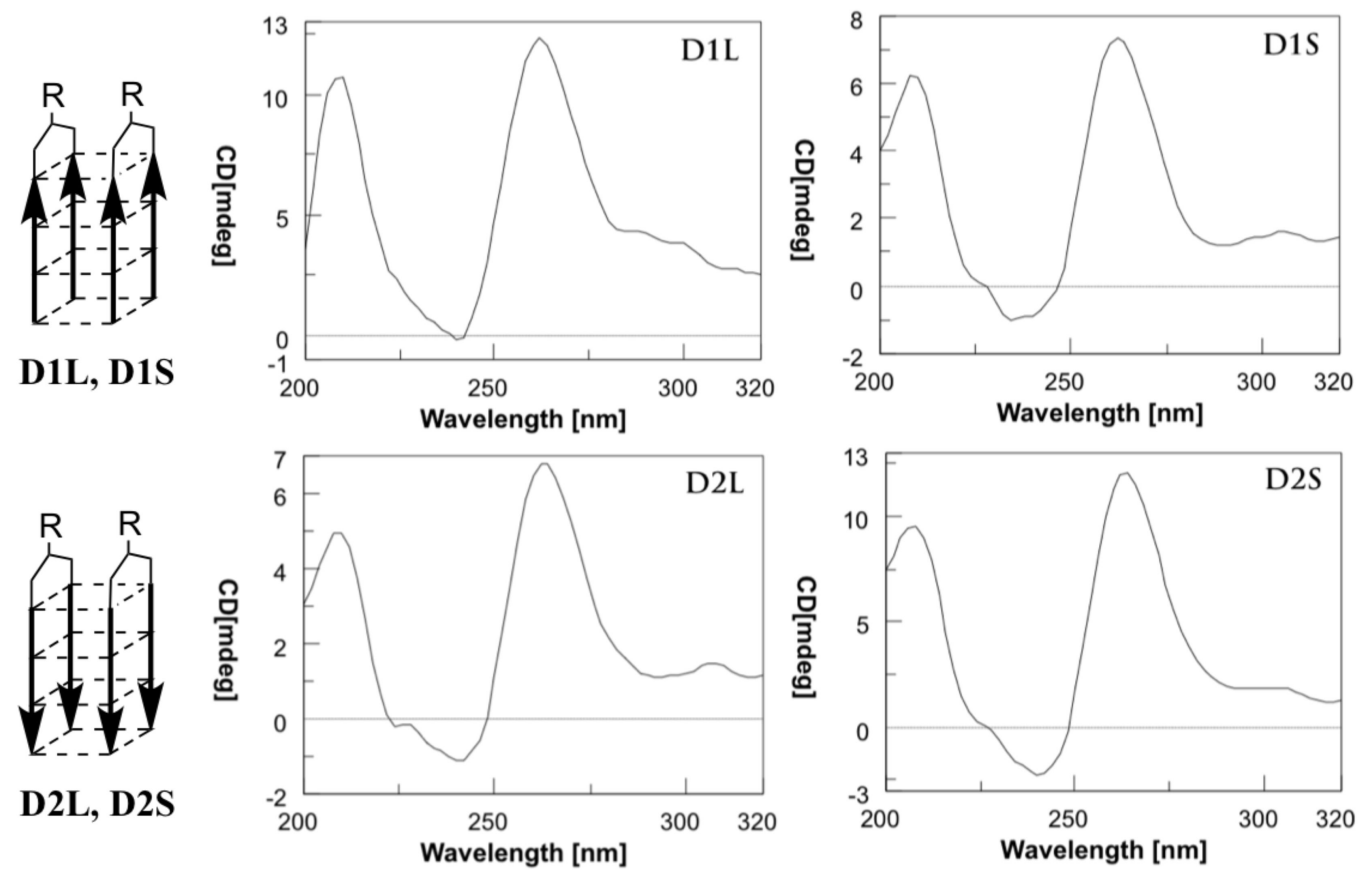
Circular dichroism spectra recorded at 5 °C of **D1L,S** and **D2L,S** annealed in 100 mM K^+^ buffer.

The results reported here demonstrate that all the synthesized DEL-(TG_4_T)_2_ fold into thermally stable, parallel quadruplexes (DEL-G4s, Figure 1), regardless of the polarity of the two ON strands and the length of the linker. Furthermore, we anticipate here that **D2L** showed a remarkable propensity to form DEL-G4 multimers with higher molecular weights, which were isolated and investigated.

## 2. Results and Discussion

### 2.1. Synthesis and Purification of DEL-(TG_4_T)_2_ (**D1L,S** and **D2L,S**)

For the synthesis of **D1L,S** and **D2L,S** (Scheme 1), we used the solid-phase phosphoramidite chemistry protocols previously optimized by us for the synthesis of TEL-ODNs [47,48,49]. The synthetic strategy uses the commercially available controlled pore glass (CPG)-sulphone-resin **1** (Scheme 1) and the phosphoramidite linkers **4** and **5** (Scheme 1), having symmetrical dimethoxytrityl (DMT)-protected ends and differing for the length of the alkyl arms to obtain the bifunctional supports **2**. Starting from **2**, **D1L,S** and **D2L,S** were obtained using 3′ or 5′ nucleotide phosphoramidite building blocks, respectively. After detachment from the solid support, the crude ON material was purified by HPLC and analyzed by analytical techniques. Support **1** was also used to obtain the tetrabranched support **3**, from which **TEL1L,S** and **TEL2L,S** were obtained following the previously reported strategy [47,48,49]. 

### 2.2. CD and CD Thermal Analyses

CD spectroscopy is a well-established technique that provides essential information on the formation and topology of G4s. The greater part of published reports indicates that parallel G4s exhibit a maximum at around 265 nm and a minimum at around 240 nm, whereas the antiparallel ones display a maximum at around 295 nm and a minimum at around 260 nm [54,55,56,57]. All G4s analysed in this study were obtained by (i) dissolving **D1L,S** and **D2L,S** in 100 mM Na^+^- or K^+^-containing buffer at 1.0 mM ON concentration, (ii) heating the solutions at 95 °C for 5 min and finally (iii) by rapidly cooling them at 4 °C. All samples were stored at 4° C for 24 h before analyses. In Figure 1, we report the CD profiles recorded at 5 °C of **D1L,S** and **D2L,S** dissolved in 100 mM K^+^ buffer. We observed similar profiles for the four DEL-ON species, with a well-defined positive maximum centred at 263 nm and a negative minimum centred at 240 nm. These data indicate that in K^+^ buffer **D1L,S** and **D2L**,**S** prevalently form parallel-oriented G4 structures independently of the orientation of the ON strands and the length of the linker. This behaviour was already observed for the TEL-(TG_4_T)_4_ G-quadruplexes, which showed CD profiles almost superimposable with those reported in Figure 1 [49,50]. The CD spectra of **D1L**,**S** and **D2L**,**S** recorded at 5° in 100 mM Na^+^ buffer (Supplementary Materials, Figure S1), essentially matched those obtained in K^+^ buffer. Taken together, the CD data of all DEL-(TG_4_T)_2_ analogues account for the formation of parallel, bimolecular DEL-G4s, both in Na^+^- and K^+^-containing buffer (Figure 1). This hypothesis was also confirmed by the PAGE and HPLC–SEC analyses (see below) that confirmed the bimolecularity of the main complex formed by all DEL-G4s. Furthermore, to evaluate the influence of DEL moiety on the stability of the resulting DEL-G4s, CD thermal denaturation experiments were performed to monitor the CD_263 nm_ value in the temperature range 5–80 °C at a heating rate of 0.5 °C/min. In 100 mM K^+^ buffer, all DEL-G4s were stable up to 80 °C, as indicated by the irrelevant variation of the CD_263 nm_ values up to this temperature. On the contrary, in the presence of 100 mM Na^+^ buffer, the increase in temperature led to a significant reduction of the CD_263 nm_ value and reliable melting temperatures (T_½_, Table 1) were calculated from the resulting sigmoidal melting curves (Figure S2). These data indicated that DEL-G4s formed by **D1L** and **D1S** have almost the same T_½_ values (55 and 54 °C, respectively) as their tetramolecular counterpart (TG_4_T)_4_ (58 °C) recorded in the same conditions. On the other hand, the T_½_ values of DEL-G4s formed by **D2L** and **D2S** (64 °C for both) were about 10 °C higher than those of **D1L** and **D1S**, indicating that the attachment of the DEL moiety at the 5′ end of the TG_4_T strand results in DEL-G4s provided with higher thermal stability. Furthermore, the invariance of T_½_ values between DEL-G4s incorporating either the long or short DEL disclosed that the length of the DEL arms does not affect the thermal stability of the resulting DEL-G4s. 

### 2.3. Electrophoretic Gel Mobility Studies

**Figure 2:**
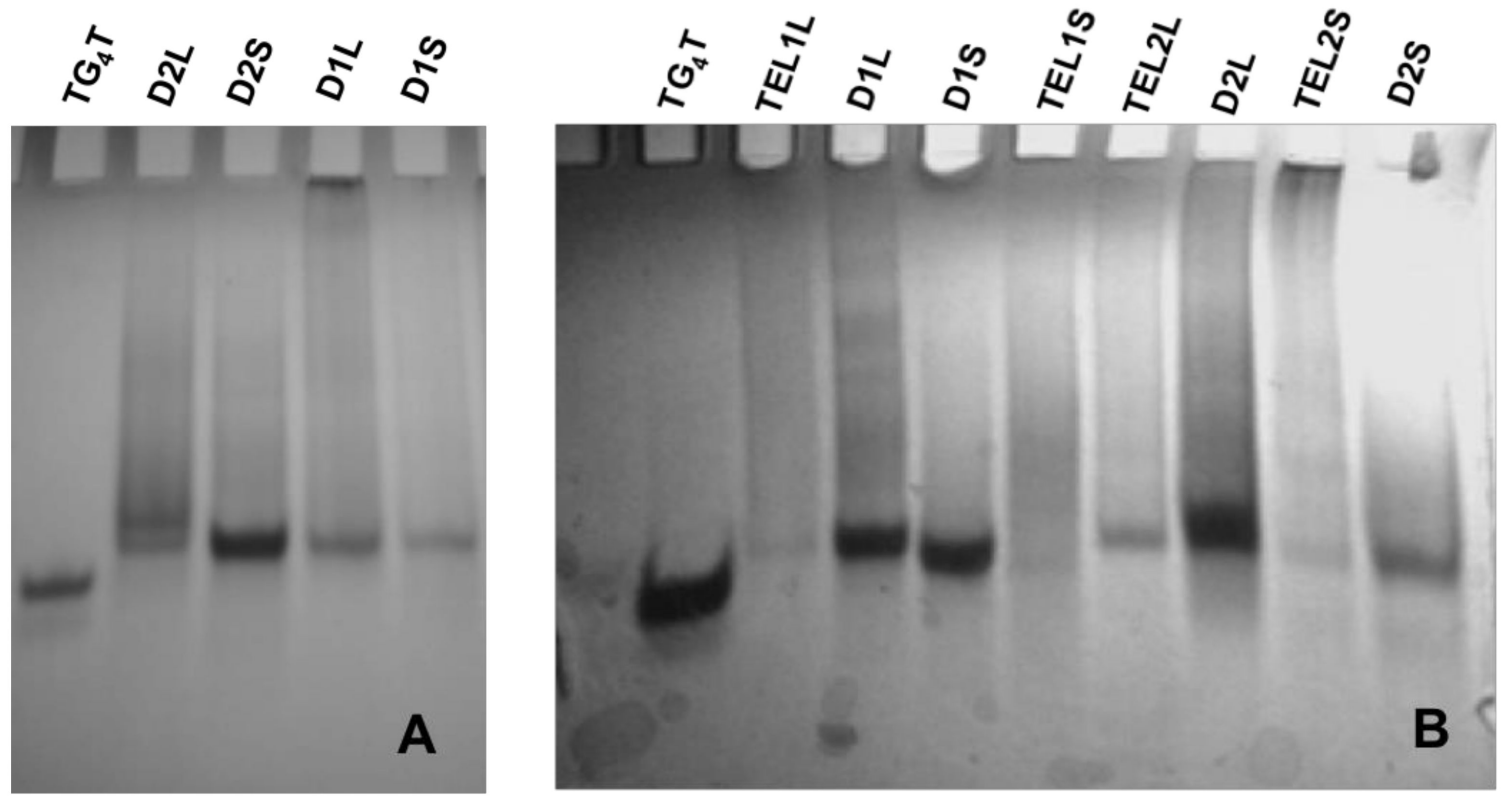
PAGE analyses of complexes formed by DEL-(TG_4_T)_2_ ODNs (**D1L,S** and **D2L,S**) in comparison with the (TG_4_T)_4_ G4 (**A**) and with the complexes formed by TEL-(TG_4_T)_4_ ODNs (**TEL1L,S** and **TEL2L,S**) (**B**). All samples were annealed in 100 mM K^+^ buffer.

To obtain information about the propensity of DEL-ODNs to fold into DEL-G4s and also on the molecularity of DEL-G4s, we performed PAGE analyses of **D1L,S** and **D2L,S**. Figure 2A displays the electrophoretic gel mobility of each complex in comparison with that of the tetramolecular G4 formed by TG_4_T. All DEL-(TG_4_T)_2_ ODNs showed a very similar PAGE running behaviour, characterized by an intense, well-defined band which migrated slightly slower than the band of the (TG_4_T)_4_ G-quadruplex and by a consistent smearing phenomenon (Figure 2A). This behaviour suggests that all DEL-ODNs formed mainly the bimolecular G4 complexes depicted in Scheme 1 and that the presence of traces of higher MW complexes cannot be ruled out. The slight reduction in the mobility of the main band can be attributed to the presence of the two DEL linkers in each DEL-G4 complex. In the PAGE reported in Figure 2B, the mobility of the complexes formed by DEL-ODNs was compared to that of TEL-ODNs depicted in Scheme 1. DEL and TEL complexes produced bands having almost the same mobility and both produced excessive smearing. The smearing is considerably marked for **D1L** and **D2L** and additional slower bands are visible. Conversely, the DEL-ODNs which were built using the short linker (**D1S** and **D2S**) migrated as better-defined bands and with a reduced smearing phenomenon. Taken together, the PAGE analyses indicated that: (i) all the here-presented DEL-ODNs fold primarily into the target bimolecular parallel DEL-G4s depicted in Scheme 1; (ii) the consistent smearing observed for all DEL and TEL complexes can be attributed to the presence of additional supramolecular structures (likely based on parallel G4 scaffolds, in agreement with CD and NMR evidence); (iii) **D1L** and **D2L** incorporating the longer linker have the highest propensity to form supramolecular G4 species.

### 2.4. Size-Exclusion Chromatography Studies

**Figure 3:**
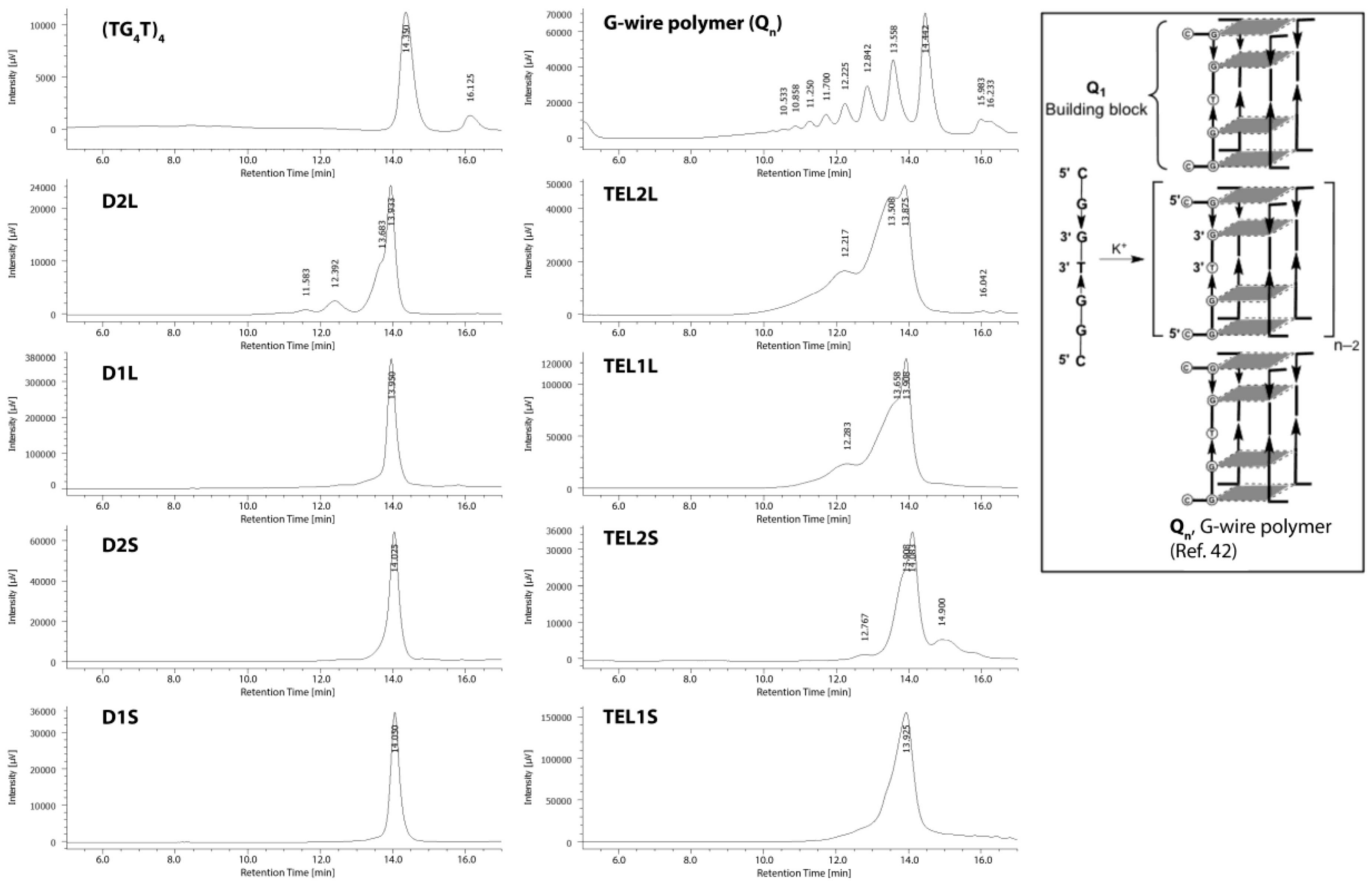
HPLC–SEC chromatograms of the complexes formed by DEL-(TG_4_T)_2_ (**D1L**,**S**, **D2L**,**S**) and TEL-(TG_4_T)_4_ (**TEL1L**,**S** and **TEL2L**,**S**) in comparison with the (TG_4_T)_4_ G4 (Q_1_) and with the Q_n_ G-wires.

**Figure 4:**
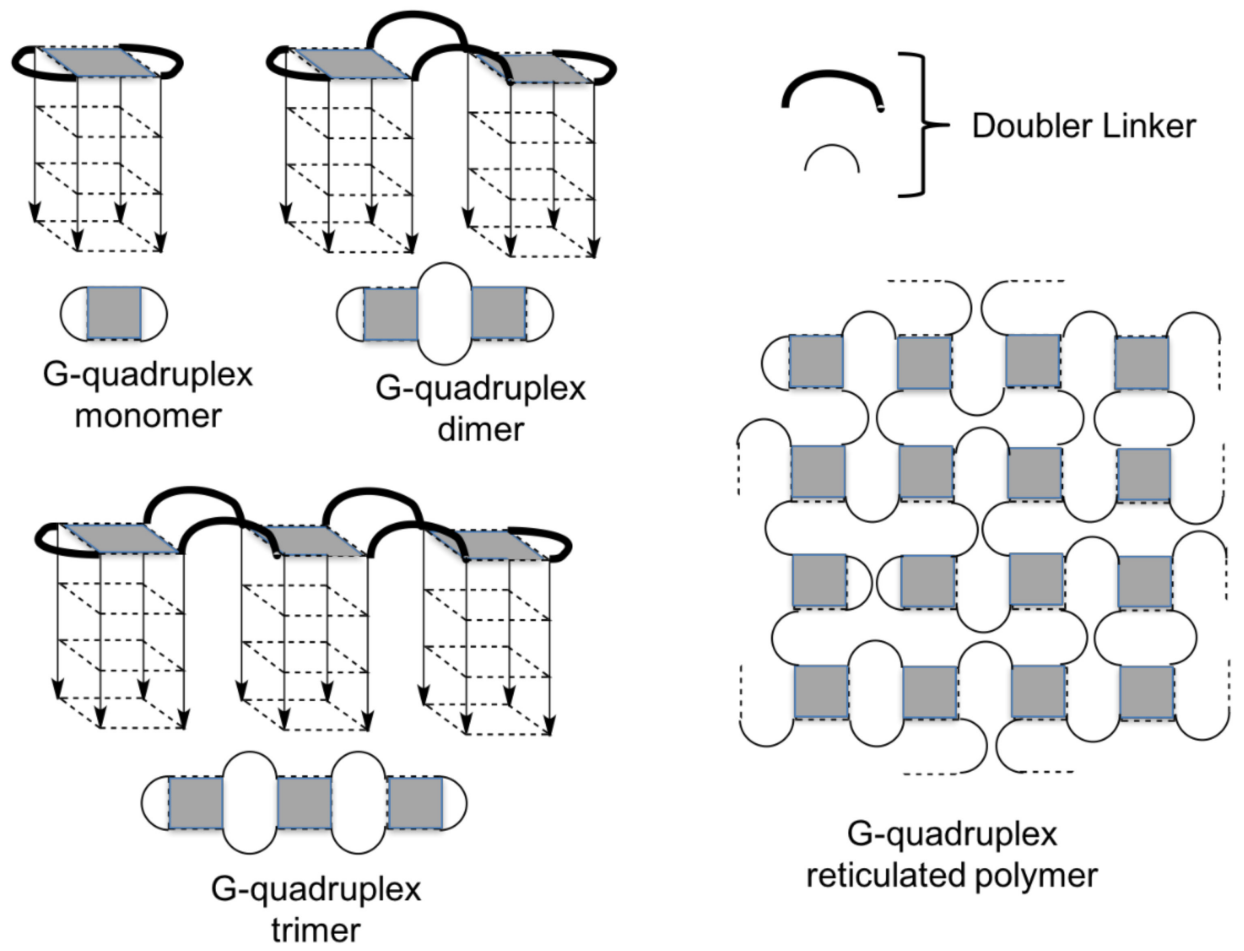
Schematic representation of the parallel G4s obtainable from the self-assembly of DEL-ODNs. The first three complexes, having the least MW, are shown on the left. The hypothetical topology of G4 supramolecular nanostructures based on reticulated parallel DEL-G4s is shown on the right.

After annealing in 100 mM K^+^ buffer, DEL- and TEL-ODNs were also analysed by HPLC–SEC at room temperature (Figure 3) with the aim of further investigating the molecularity of the resulting complexes. The tetramolecular G4 formed by the TG_4_T sequence (see (TG_4_T)_4_ in Scheme 1) and the G4 multimers formed by the ^5′^CGG^3′^-^3′^TGGC^5′^ sequence (Q_n_ in Figure 3) [42] were used as size markers for monomeric (Q_1_) and multimeric G4 building blocks (Q_2–n_), respectively. The HPLC–SEC analysis of both size markers indicated that in the used column and conditions the monomeric Q_1_ scaffolds (containing 24 or 28 nucleotides, respectively) had retention times (t_R_) in the range 14.3–14.5 min, and the Q_n_ multimers had t_R_ lower than 13.5 min (corresponding to the Q_2_ complex for the Q_n_ marker). The HPLC–SEC analysis of DEL-ODNs analogues confirmed that they fold primarily into a Q_1_ complex (t_R_ centred at 14.00 min). The slightly reduced t_R_ observed for DEL-G4s compared to those of the Q_1_ size markers can be attributed to the presence of the two DEL moieties in the Q_1_-like DEL-G4 complexes. Differently from what observed by PAGE, the HPLC–SEC profiles of all but **D2L** DEL-ODNs did not show additional peaks attributable to higher MW complexes, such as DEL-G4 multimers or other polymeric species. As anticipated, only **D2L** showed an intense shoulder peak at t_R_ 13.68 min as well as a defined peak eluted at min 12.38, likely attributable to DEL-G4 multimers as those depicted in Figure 4. 

The t_R_ of all peaks corresponding to TEL-G4 monomers (Q_1_), obtained by performing the HPLC–SEC separation of the annealed TEL-ODNs (Figure 3, right column), were almost superimposable with those of the corresponding peaks obtained from the annealed DEL-ODNs. This result indicated that the size of the linker and the polarity of the linkages have a negligible effect on the t_R_ of the corresponding DEL- and TEL-G4s, thus implying that the two classes of modified G4s have a similar 3D conformation. However, the comparison of the whole HPLC–SEC profiles evidenced a higher propensity of all TEL-ODNs to form higher molecular weight (MW) complexes (in particular for **TEL1L** and **TEL2L**), probably due to more favourable entropic parameters. 

### 2.5. ^1^H-NMR Studies

The formation of G4s from G-rich oligonucleotides and their analogues is usually confirmed by the observation in the downfield region (10–13 ppm) of water-suppressed ^1^H-NMR spectra of signals belonging to the exchange-protected N-1 imino protons of guanosines involved in the formation of G-tetrads. When guanosines are involved in the formation of a G-tetrad, their N-1 imino proton is engaged in the formation of a Hoogsteen-type hydrogen bond with the O-6 oxygen atom of flanking guanosine and does not exchange with the protons of bulk water [54,58]. For example, the water-suppressed ^1^H-NMR spectrum of the highly symmetric (TG_4_T)_4_ quadruplex is characterized by the presence of four sharp imino proton signals, one signal for each of the four G-tetrads (Figure S3) [52,53], whereas four signals are expected for antiparallel and 3+1 mixed G4s [59].

**Figure 5:**
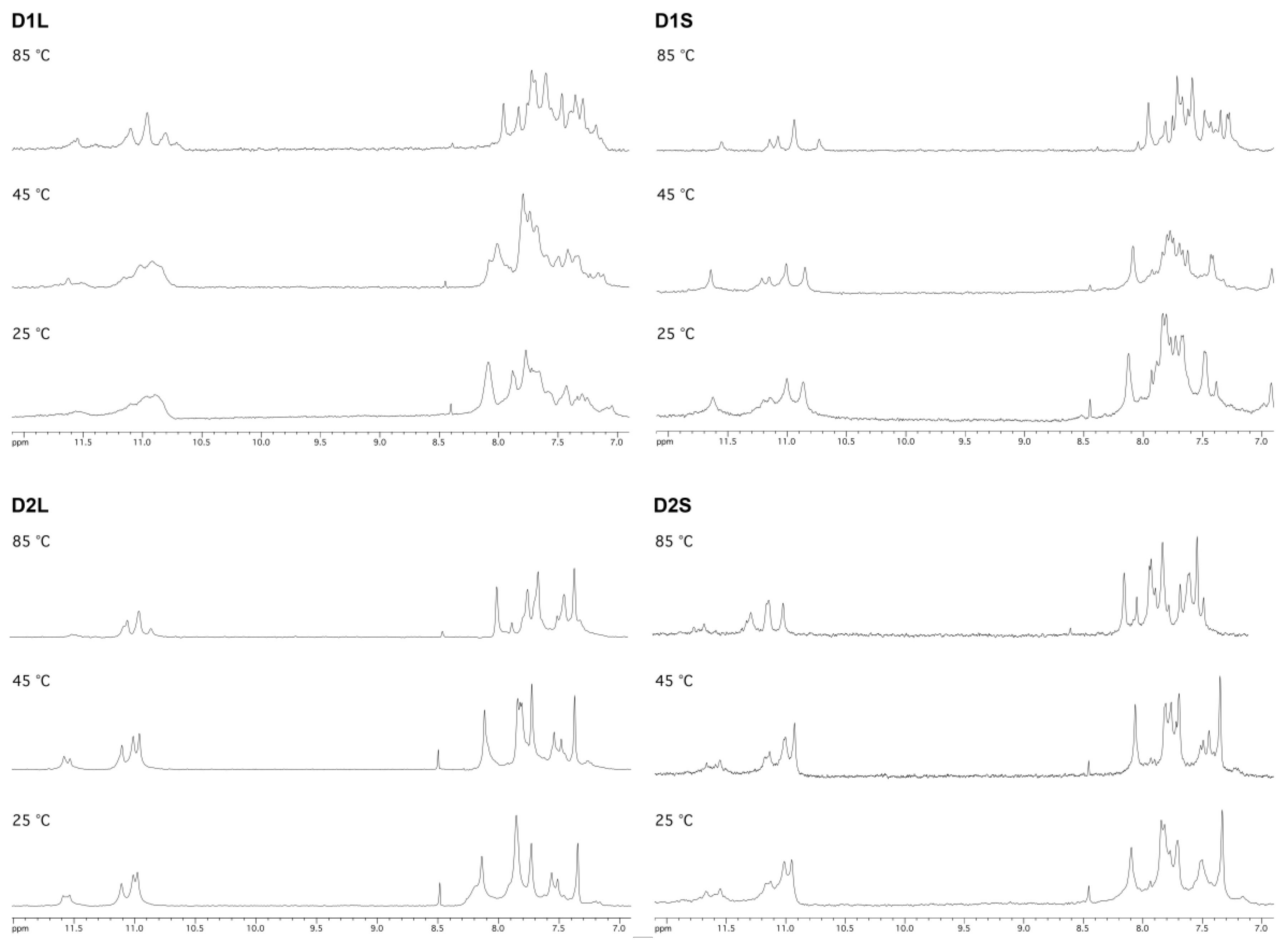
Downfield region of the water-suppressed NMR spectra of **D1L,S** and **D2L,S** annealed in 100 mM K^+^ buffer and recorded at 25, 45 and 85 °C.

The aromatic and imino protons region of the water-suppressed ^1^H-NMR spectra recorded at 25, 45 and 85 °C of the studied DEL-ODNs are reported in Figure 5. The presence of strong imino proton signals (10.7–11.8 ppm) in all recorded spectra confirmed that the four new DEL-ODNs fold into DEL-G4s when annealed in K^+^-containing buffer. The imino proton regions of the NMR spectra of the four DEL-ODNs were very similar to that of the tetramolecular parallel (TG_4_T)_4_ G4 (Figure S3) and those of the corresponding TEL-G4s [49], both concerning the number and the resonance frequencies of the imino protons peaks. These signals were detectable up to 85 °C, in agreement with the CD melting results reported in Table 1. Only in the case of **D1L** at 25 and 45 °C, we did not observe any well-resolved imino proton signal but only very broad signals resonating at the expected frequencies. This behaviour could be explained with the concomitant presence in solution of small amounts of less stable, rapidly interconverting, bimolecular G4s having different topology (e.g., antiparallel and/or 3+1), as suggested by the presence of a weak positive CD signal at around 290 nm and by the unusually weaker negative CD signal at around 240 nm (Figure 1). 

### 2.6. Isolation and Analyses of Higher Molecular Weight Species Produced by **D2L**

**Figure 6:**
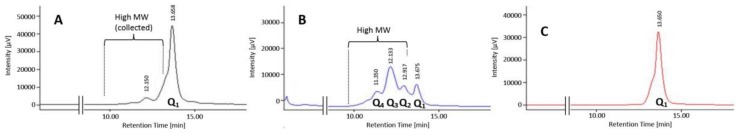
HPLC–SEC chromatograms of: (**A**) complexes formed by **D2L**; (**B**) reinjection of the higher MW species formed by **D2L** collected as shown in panel A; (**C**) reinjection of the purified DEL-Q_1_ complex formed by **D2L** collected as shown in panel A.

**Figure 7:**
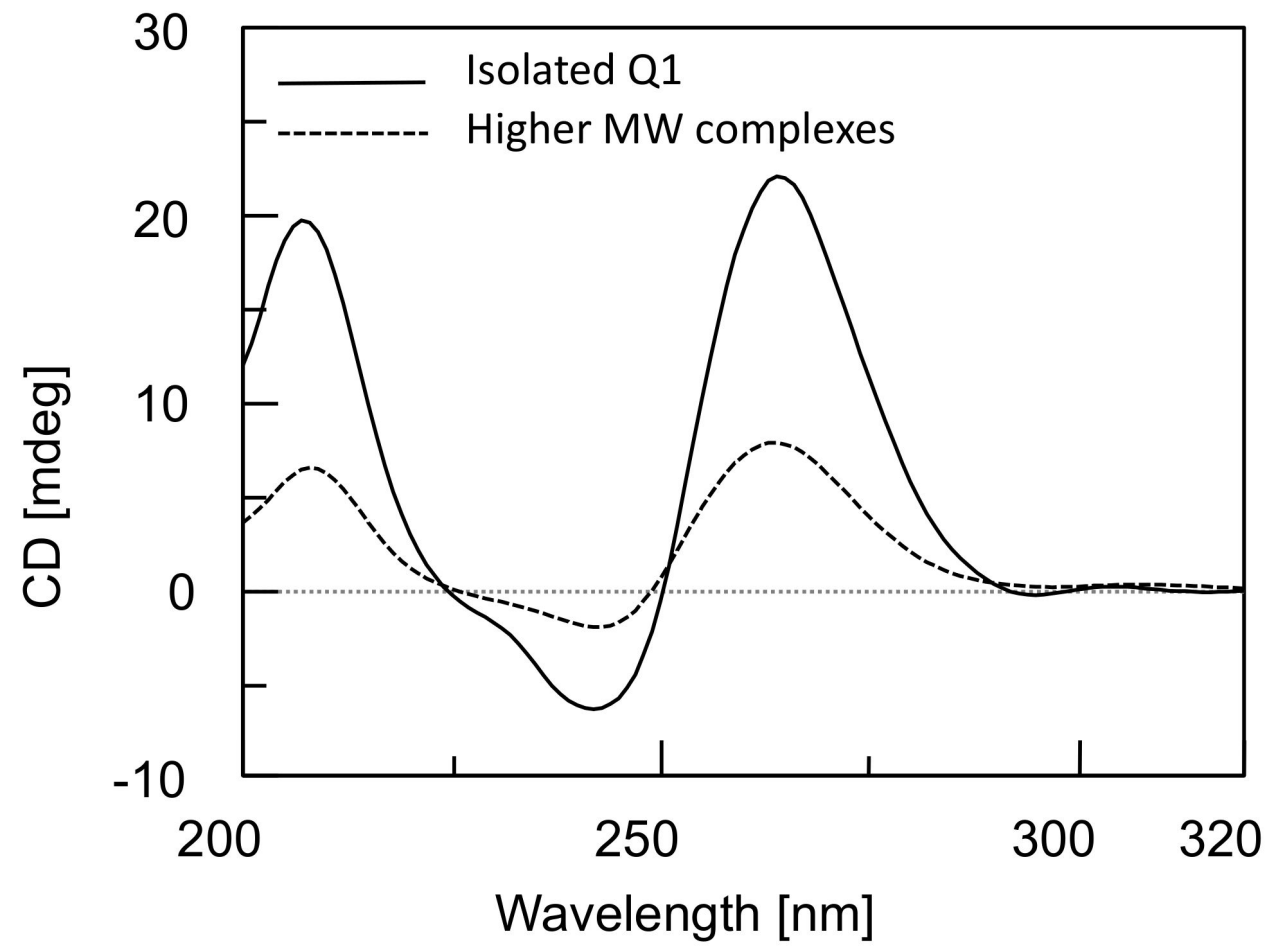
CD spectra of DEL-Q_1_ and higher MW species obtained by HPLC–SEC fractionation of **D2L** (100 mM K^+^-containing buffer, 5 °C).

To obtain further structural information on the higher MW complexes formed by **D2L**, we collected in a single fraction the least retained peaks of the HPLC–SEC fractionation of **D2L**, as indicated in Figure 6A, and reinjected them on the same column 24 h after storage at 4 °C. The resulting HPLC profile (Figure 6B) presented four partially resolved peaks, which we attributed to the bimolecular DEL-G4 (Q_1_, t_R_ = 13.7 min) and to the DEL-G4 multimers incorporating two (Q_2_, t_R_ = 12.9 min), three (Q_3_, t_R_ = 12.1 min), and four (Q_4_, t_R_ = 11.4 min) G4 scaffolds. To confirm the G4 architecture of the higher MW complexes, we collected them, as indicated in Figure 6B, and recorded their CD spectrum, which resulted almost superimposable to that of the isolated Q_1_ species (Figure 7). We attributed the small amount of Q_1_ detected in Figure 6B to the partial recovery of this species during the first HPLC–SEC fractionation and not to the dissociation of higher MW DEL-G4 multimers. This hypothesis was corroborated by the HPLC–SEC profile shown in Figure 6C (obtained by injecting the Q1 peak isolated from the first HPLC fractionation of **D2L**), which, not showing any trace of least retained peaks attributable to DEL-G4 complexes belonging to DEL-Q_>2_ types, confirmed that, once isolated, the DEL-Q_n_ multimers do not interconvert one into the other. The latter observation agrees in full with the formation of G4 multimers based on reticulated G4 building blocks (Figure 4), rather than on end-to-end stacked G4 building blocks (like the Q_n_ species reported in Figure 3). These results suggest that DEL-G4 multimers comprising more than four G4 scaffolds, similar to the G4 reticulated polymer shown in Figure 4, could also be obtainable by using higher concentrations of DEL-ONs and/or potassium ions and by optimizing the annealing procedure.

## 3. Materials and Methods

### 3.1. Reagents and Equipment

Chemicals and solvents were purchased from Sigma-Aldrich (Milan, Italy). Reagents and phosphoramidites for DNA syntheses were purchased from Glen Research (Sterling, VA, USA). The DEL linker **2**, the TEL linker **3** and all the ODNs were assembled on a PerSeptive Biosystems (Framingham, MA, USA) Expedite DNA synthesizer using standard phosphoramidite chemistry. HPLC analyses and purifications were performed with a Jasco (Jasco Europe Srl, Cremella, LC, Italy) PU2089 pump system equipped with an UV detector model 2075 Plus. CPG-resin **1** and the linkers **4** and **5** were purchased from Glen Research. UV spectra were registered using a Jasco V-530 spectrophotometer. CD spectra and thermal denaturation experiments were obtained with a Jasco 1500 CD spectrophotometer equipped with a Jasco PTC-348-WI temperature controller unit. NMR spectra were recorded either on a Varian (Palo Alto, CA, USA) Unity Inova 500 MHz spectrometer equipped with a broadband inverse probe with z-field gradient, or on a Varian Unity INOVA 700 MHz spectrometer equipped with a triple resonance cryoprobe. All NMR spectra were processed using the Varian VNMR and iNMR (http://www.inmr.net) software packages. PAGE bands were visualized on a Bio-Rad Laboratories (Segrate, MI, Italy) Gel Doc^TM^ XR+ image system. 

### 3.2. Syntheses and Purifications of DEL-ODNs (**D1L,S** and **D2L,S**)

50 mg of controlled pore glass (CPG) support **1** (0.18 mmol/g) were used for each synthesis in the automated DNA synthesizer following standard phosphoramidite chemistry. 45 mg/mL solutions of phosphoramidite **4** or **5** (both in anhydrous CH_3_CN) were used for the synthesis of **2L** or **2S**, respectively. This step was followed by reactions with 3′-phosphoramidite (for **D1L** and **D1S**) or 5′-phosphoramidite (for **D2L** and **D2S**) nucleotide building blocks (six cycles, 45 mg/mL in anhydrous CH_3_CN). The coupling yields were consistently higher than 98% (by DMT spectrophotometric measurements). The solid support was then treated with concentrated aqueous ammonia solution for 7 h at 55 °C. The filtered solution and washings were concentrated under reduced pressure and purified by HPLC with an anion exchange column (Macherey–Nagel, 1000-8/46, 4.4 × 50 mm, 5 μm) eluted with a linear gradient from 0 to 100% B in 30 min; buffer A: 20 mM NaH_2_PO_4_, pH 7.0 containing 20% CH_3_CN; buffer B: 1 M NaCl, 20 mM NaH_2_PO_4_, pH 7.0, containing 20% CH_3_CN; flow rate 1 mL/min. The collected products were desalted by gel filtration on a BioGel P2 column eluted with H_2_O/ethanol (9:1, *v*/*v*) to obtain, after lyophilisation, pure **D1L,S** and **D2L,S** (92, 85 and 80, 88 OD_260_ units, respectively). The ON concentrations were determined spectrophotometrically in water at λ = 260 nm and 90 °C using the molar extinction coefficient ε = 28,900 cm^−1^ M^−1^ for DEL-(TG_4_T)_2_ and ε = 57,800 for TEL-(TG_4_T)_4_ calculated for the unstacked ODNs by the nearest-neighbour method. 

### 3.3. Preparation of the G-Quadruplexes (Annealing)

All G4s were formed by dissolving DEL-ODNs, TEL-ODNs, or TG_4_T in the K^+^ buffer (90 mM KCl, 10 mM KH_2_PO_4_, pH 7.0) or Na^+^ buffer (90 mM NaCl, 10 mM NaH_2_PO_4_, pH 7.0) and heating to 95 °C for 5 min and then rapidly cooling at 4 °C (annealing). All samples were stored at 4 °C for 24 h before analyses. The solutions were equilibrated at 25 °C for 2 h before performing the experiments.

### 3.4. PAGE Experiments

Native gel electrophoresis experiments were performed on 12% polyacrylamide gels containing 1× TBE buffer, pH 7.0 with 30 mM KCl, at 4 °C, 120 V for 2 h. Samples were loaded at a final ON concentration of 100 µM; glycerol was added (10% final) to facilitate sample loading in the wells. The bands were finally visualized by UV shadowing or by SYBR Green staining. 

### 3.5. CD Experiments

CD spectra and CD melting profiles were recorded in 0.1 cm optical path quartz cuvettes (100 nm/min scanning speed, 1 s response time). The spectra were recorded in triplicate at 5 °C from 200 to 320 nm. CD samples were prepared in the above reported 0.1 M K^+^ or Na^+^ buffer at the final single-strand concentration of 20 µM. The buffer baseline was subtracted from each spectrum. The CD melting experiments were performed by monitoring the CD value (mdeg) at 263 nm in the temperature range 5–95 °C at 0.5 °C/min heating rate.

### 3.6. HPLC–SEC Analyses

HPLC–SEC analyses and purifications were performed using a Phenomenex (Bologna, Italy) Yarra SEC-2000 column (300 × 7.8 mm, 3 µm) eluted with 90 mM KCl and 10 mM KH_2_PO_4_/CH_3_CN (80:20, *v*/*v*), flow rate 0.6 mL/min, UV-detector at 260 nm. The analyses were performed at room temperature. 

### 3.7. NMR Experiments

NMR samples were prepared at 1.8 mM single strand concentration in 200 mL of 100 mM K^+^ solution (90 mM KCl, 10 mM KH_2_PO_4_, pH 7.0, 9:1 H_2_O/D_2_O). Water suppression was achieved by including a double pulsed-field gradient spin-echo (DPFGSE) module [60,61] in the pulse sequence prior to acquisition. NMR spectra were acquired as 16,384 data points with a recycle delay of 1.0 s at 25, 45, and 85 °C, and the spectra were apodized with a shifted sine bell squared window function.

## 4. Conclusions

In this study, we reported the synthesis and the structural characterization of a new kind of G4 forming G-rich oligonucleotides, named DEL-ODNs, in which two TG_4_T strands are attached either by their 5′ or 3′ end to the two ends of a bifunctional linker. A total of four DEL-(TG_4_T)_2_ ODNs were synthesized using two linkers of different length, and their propensity to form DEL-G4s was investigated by CD, NMR, PAGE and HPLC–SEC analyses. CD and NMR spectroscopies confirmed the formation of the target bimolecular parallel G4s for all the four DEL-ODNs, regardless of the polarity of the two ON strands and the length of the linker. All four DEL-G4s were provided with extremely high thermal stability, especially in 100 mM K^+^-containing solution (T_½_ > 80 °C). The here-described DEL-G4s were also compared with the corresponding G4s formed by the unmodified TG_4_T sequence and by the previously reported TEL-(TG_4_T)_4_ analogues, with the aim of adding useful information on the effect of DEL and TEL linkers on the formation of supramolecular structures based on the parallel (TG_4_T)_4_ G4 scaffold. HPLC–SEC and PAGE analyses, used to obtain information on the molecularity of the complexes, confirmed that all DEL-(TG_4_T)_2_ ODNs formed the target bimolecular DEL-G4s primarily. However, detectable amounts of higher molecular-weight species were detected, especially for **D2L**, which was obtained by linking the 5′ end of two TG_4_T strands to the longer linker. Looking at the whole picture, our results indicate that: i) TEL-ODNs have a higher propensity to fold into higher MW G4 complexes than the corresponding DEL-ODNs, likely because of the entropy gain; ii) DEL- and TEL-ODNs incorporating the longer linker have the higher propensity to fold into multimeric G4 complexes; iii) the direction of the TG_4_T synthesis on the four TEL arms plays only a marginal role on the molecularity of the resulting TEL-G4s, whereas for DEL-ODNs, clear evidence of the formation of multimeric DEL-G4s was observed just for **D2L**. At the studied concentration of **D2L** and potassium ions, the formation of higher MW G4 complexes comprising up to four parallel G4 scaffolds was confirmed by HPLC–SEC. Taken together, the here-reported results suggest that the synthetic strategy based on DEL and TEL linkers could be further exploited to obtain new supramolecular biomaterials based on the reticulated G4 scaffolds depicted in Figure 4.

## Figures and Tables

**Table 1:** Melting temperatures (T_½_ values in °C) of DEL-(TG_4_T)_2_ and TG_4_T ODNs annealed in 100 mM K^+^- or Na^+^-containing buffer (λ = 263 nm, 0.5 °C/min heating rate).

ODN	T_½_ (K^+^)	T_½_ (Na^+^)
**D1L**	>80	55
**D1S**	>80	54
**D2L**	>80	64
**D2S**	>80	64
**TG_4_T**	>80	58

## Supplementary Materials

The following are available online, Figure S1: CD spectra of samples annealed in 100 mM Na^+^-containing buffer, Figure S2: CD melting curves of samples annealed in 100 mM Na+-containing buffer, Figure S3: imino protons region of ^1^H-NMR spectrum of (TG_4_T)_4_ G-quadruplex.

## References

[1] Phan A.T.T., Kuryavyi V., Luu K.N., Patel D.J., Neidle S., Balasubramanian S. (2006). Structural Diversity of G-Quadruplex Scaffolds. Quadruplex Nucleic Acids.

[2] Simonsson T. (2001). G-quadruplex DNA structures variations on a theme. Biol. Chem..

[3] Parkinson G.N., Neidle S., Balasubramanian S. (2006). Fundamentals of Quadruplex Structures. Quadruplex Nucleic Acids.

[4] Searle M.S., Williams H.E.L., Gallagher C.T., Grant R.J., Stevens M.F.G. (2004). Structure and K^+^ ion-dependent stability of a parallel-stranded DNA quadruplex containing a core A-tetrad. Org. Biomol. Chem..

[5] Guédin A., De Cian A., Gros J., Lacroix L., Mergny J.-L.L. (2008). Sequence effects in single-base loops for quadruplexes. Biochimie.

[6] Risitano A., Fox K.R. (2004). Influence of loop size on the stability of intramolecular DNA quadruplexes. Nucleic Acids Res..

[7] Rachwal P.A., Findlow I.S., Werner J.M., Brown T., Fox K.R. (2007). Intramolecular DNA quadruplexes with different arrangements of short and long loops. Nucleic Acids Res..

[8] Hazel P., Huppert J., Balasubramanian S., Neidle S. (2004). Loop-length-dependent folding of G-quadruplexes. J. Am. Chem. Soc..

[9] Maizels N. (2006). Dynamic roles for G4 DNA in the biology of eukaryotic cells. Nat. Struct. Mol. Biol..

[10] Lipps H.J., Rhodes D. (2009). G-quadruplex structures: In vivo evidence and function. Trends Cell Biol..

[11] Biffi G., Tannahill D., McCafferty J., Balasubramanian S. (2013). Quantitative visualization of DNA G-quadruplex structures in human cells. Nat. Chem..

[12] Sengupta P., Basu S., Soni S., Pandey A., Roy B., Oh M.S., Chin K.T., Paraskar A.S., Sarangi S., Connor Y. (2012). Cholesterol-tethered platinum II-based supramolecular nanoparticle increases antitumor efficacy and reduces nephrotoxicity. Proc. Natl. Acad. Sci..

[13] Falanga A.P., Cerullo V., Marzano M., Feola S., Oliviero G., Piccialli G., Borbone N. (2019). PNA-functionalized adenoviral vectors targeting G-quadruplexes in the P1 promoter of Bcl-2 proto-oncogene: A new tool for gene modulation in anti-cancer therapy. Bioconjug. Chem..

[14] Xu Y. (2011). Chemistry in human telomere biology: Structure, function and targeting of telomere DNA/RNA. Chem. Soc. Rev..

[15] Patel D.J., Phan A.T.T., Kuryavyi V. (2007). Human telomere, oncogenic promoter and 5′-UTR G-quadruplexes: Diverse higher order DNA and RNA targets for cancer therapeutics. Nucleic Acids Res..

[16] Avino A., Fabrega C., Tintore M., Eritja R. (2012). Thrombin binding aptamer, more than a simple aptamer: Chemically modified derivatives and biomedical applications. Curr. Pharm. Des..

[17] Aaldering L.J., Poongavanam V., Langkjær N., Murugan N.A., Jørgensen P.T., Wengel J., Veedu R.N. (2017). Development of an Efficient G-Quadruplex-Stabilised Thrombin-Binding Aptamer Containing a Three-Carbon Spacer Molecule. ChemBioChem.

[18] Politi J., Rea I., Nici F., Dardano P., Terracciano M., Oliviero G., Borbone N., Piccialli G., De Stefano L. (2016). Nanogravimetric and optical characterizations of thrombin interaction with a self-assembled thiolated aptamer. J. Sensors.

[19] Scuotto M., Persico M., Bucci M., Vellecco V., Borbone N., Morelli E., Oliviero G., Novellino E., Piccialli G., Cirino G. (2014). Outstanding effects on antithrombin activity of modified TBA diastereomers containing an optically pure acyclic nucleotide analogue. Org. Biomol. Chem..

[20] Borbone N., Bucci M., Oliviero G., Morelli E., Amato J., D’Atri V., D’Errico S., Vellecco V., Cirino G., Piccialli G. (2012). Investigating the Role of T 7 and T 12 Residues on the Biological Properties of Thrombin-Binding Aptamer: Enhancement of Anticoagulant Activity by a Single Nucleobase Modi fi cation. J. Med. Chem..

[21] Hotoda H., Momota# K., Furukawa# H., Nakamuta$ T., Kaneko M., Kimura S., Shimada K. (1994). Biologically active oligodeoxyribonucleotides—II^1^: Structure activity relationships of anti-HIV-1 pentadecadeoxyribonucleotides bearing 5′-end-modifications. Nucleosides Nucleotides.

[22] Wyatt J.R., Vickers T.A., Roberson J.L., Buckheit R.W., Klimkait T., DeBaets E., Davis P.W., Rayner B., Imbach J.L., Ecker D.J. (1994). Combinatorially selected guanosine-quartet structure is a potent inhibitor of human immunodeficiency virus envelope-mediated cell fusion. Proc. Natl. Acad. Sci..

[23] Oliviero G., Stornaiuolo M., D’Atri V., Nici F., Yousif A.M., D’Errico S., Piccialli G., Mayol L., Novellino E., Marinelli L. (2016). Screening Platform toward New Anti-HIV Aptamers Set on Molecular Docking and Fluorescence Quenching Techniques. Anal. Chem..

[24] Nici F., Oliviero G., Falanga A.P., D’Errico S., Marzano M., Musumeci D., Montesarchio D., Noppen S., Pannecouque C., Piccialli G. (2018). Anti-HIV activity of new higher order G-quadruplex aptamers obtained from tetra-end-linked oligonucleotides. Org. Biomol. Chem..

[25] D’Atri V., Oliviero G., Amato J., Borbone N., D’Errico S., Mayol L., Piccialli V., Haider S., Hoorelbeke B., Balzarini J. (2012). New anti-HIV aptamers based on tetra-end-linked DNA G-quadruplexes: Effect of the base sequence on anti-HIV activity. Chem. Commun..

[26] Oliviero G., Amato J., Borbone N., D’Errico S., Galeone A., Mayol L., Haider S., Olubiyi O., Hoorelbeke B., Balzarini J. (2010). Tetra-end-linked oligonucleotides forming DNA G-quadruplexes: A new class of aptamers showing anti-HIV activity. Chem. Commun..

[27] Livshits G.I., Stern A., Rotem D., Borovok N., Eidelshtein G., Migliore A., Penzo E., Wind S.J., Di Felice R., Skourtis S.S. (2014). Long-range charge transport in single G-quadruplex DNA molecules. Nat. Nanotechnol..

[28] Liu S.-P., Weisbrod S.H., Tang Z., Marx A., Scheer E., Erbe A. (2010). Direct measurement of electrical transport through G-quadruplex DNA with mechanically controllable break junction electrodes. Angew. Chemie Int. Ed..

[29] Cohen H., Sapir T., Borovok N., Molotsky T., Di Felice R., Kotlyar A.B., Porath D. (2007). Polarizability of G4-DNA observed by electrostatic force microscopy measurements. Nano Lett..

[30] Ruttkay-Nedecky B., Kudr J., Nejdl L., Maskova D., Kizek R., Adam V. (2013). G-quadruplexes as sensing probes. Molecules.

[31] Yang D., Campolongo M.J., Nhi Tran T.N., Ruiz R.C.H., Kahn J.S., Luo D. (2010). Novel DNA materials and their applications. Wiley Interdiscip. Rev. Nanomed. Nanobiotechnol..

[32] Linko V., Dietz H. (2013). The enabled state of DNA nanotechnology. Curr. Opin. Biotechnol..

[33] Rothemund P.W.K. (2006). Folding DNA to create nanoscale shapes and patterns. Nature.

[34] Marsh T.C., Vesenka J., Henderson E. (1995). A new DNA nanostructure, the G-wire, imaged by scanning probe microscopy. Nucleic Acids Res..

[35] Construction and Examination of “G-Wire” DNA. https://aip.scitation.org/doi/abs/10.1063/1.1520082.

[36] Marsh T.C., Henderson E. (1994). G-Wires: Self-Assembly of a Telomeric Oligonucleotide, d(GGGGTTGGGG), into Large Superstructures. Biochemistry.

[37] Kotlyar A.B., Borovok N., Molotsky T., Cohen H., Shapir E., Porath D. (2005). Long, monomolecular guanine-based nanowires. Adv. Mater..

[38] Shi Y., Luo H.Q., Li N.B. (2013). A highly sensitive resonance Rayleigh scattering method to discriminate a parallel-stranded G-quadruplex from DNA with other topologies and structures. Chem. Commun..

[39] Saintomé C., Amrane S., Mergny J.-L.L., Alberti P. (2016). The exception that confirms the rule: A higher-order telomeric G-quadruplex structure more stable in sodium than in potassium. Nucleic Acids Res..

[40] Smargiasso N., Rosu F., Hsia W., Colson P., Baker E.S., Bowers M.T., De Pauw E., Gabelica V. (2008). G-quadruplex DNA assemblies: Loop length, cation identity, and multimer formation. J. Am. Chem. Soc..

[41] Borbone N., Amato J., Oliviero G., D’Atri V., Gabelica V., De Pauw E., Piccialli G., Mayol L. (2011). D(CGGTGGT) forms an octameric parallel G-quadruplex via stacking of unusual G(:C):G(:C):G(:C):G(:C) octads. Nucleic Acids Res..

[42] Oliviero G., D’Errico S., Pinto B., Nici F., Dardano P., Rea I., De Stefano L., Mayol L., Piccialli G., Borbone N. (2017). Self-assembly of g-rich oligonucleotides incorporating a 3′-3′ inversion of polarity site: A new route towards G-Wire DNA nanostructures. ChemistryOpen.

[43] D’Atri V., Borbone N., Amato J., Gabelica V., D’Errico S., Piccialli G., Mayol L., Oliviero G., D’Atri V., Borbone N. (2014). DNA-based nanostructures: The effect of the base sequence on octamer formation from d(XGGYGGT) tetramolecular G-quadruplexes. Biochimie.

[44] Ferreira R., Alvira M., Aviñó A., Gómez-Pinto I., González C., Gabelica V., Eritja R. (2012). Synthesis and structural characterization of stable branched DNA G-quadruplexes using the trebler phosphoramidite. ChemistryOpen.

[45] Murat P., Bonnet R., Van Der Heyden A., Spinelli N., Labbé P., Monchaud D., Teulade-Fichou M.P., Dumy P., Defrancq E. (2010). Template-Assembled Synthetic G-Quadruplex (TASQ): A Useful System for Investigating the Interactions of Ligands with Constrained Quadruplex Topologies. Chem.—A Eur. J..

[46] Murat P., Cressend D., Spinelli N., Van Der Heyden A., Labbé P., Dumy P., Defrancq E. (2008). A novel conformationally constrained parallel G quadruplex. ChemBioChem.

[47] Oliviero G., Borbone N., Galeone A., Varra M., Piccialli G., Mayol L. (2004). Synthesis and characterization of a bunchy oligonucleotide forming a monomolecular parallel quadruplex structure in solution. Tetrahedron Lett..

[48] Oliviero G., Amato J., Borbone N., Galeone A., Varra M., Piccialli G., Mayol L. (2006). Synthesis and characterization of DNA quadruplexes containing T-tetrads formed by bunch-oligonucleotides. Biopolymers.

[49] Oliviero G., Amato J., Borbone N., Galeone A., Petraccone L., Varra M., Piccialli G., Mayol L. (2006). Synthesis and characterization of monomolecular DNA G-quadruplexes formed by tetra-end-linked oligonucleotides. Bioconjug. Chem..

[50] Oliviero G., Borbone N., Amato J., D’Errico S., Galeone A., Piccialli G., Varra M., Mayol L. (2009). Synthesis of quadruplex-forming tetra-end-linked oligonucleotides: Effects of the linker size on quadruplex topology and stability. Biopolymers.

[51] Aviño A., Grimau M.G., Frieden M., Eritja R. (2004). Synthesis and Triple-Helix-Stabilization Properties of Branched Oligonucleotides Carrying 8-Aminoadenine Moieties. Helv. Chim. Acta.

[52] Aboul-ela F., Murchie A.I.H., Lilley D.M.J. (1992). NMR study of parallel-stranded tetraplex formation by the hexadeoxynucleotide d(TG4T). Nature.

[53] Mergny J.-L.L., De Cian A., Ghelab A., Saccà B., Lacroix L. (2005). Kinetics of tetramolecular quadruplexes. Nucleic Acids Res..

[54] Jin R., Gaffney B.L., Wang C., Jones R.A., Breslauer K.J. (1992). Thermodynamics and structure of a DNA tetraplex: a spectroscopic and calorimetric study of the tetramolecular complexes of d(TG3T) and d(TG3T2G3T). Proc. Natl. Acad. Sci. USA.

[55] Hardin C.C., Perry A.G., White K. (2000). Thermodynamic and kinetic characterization of the dissociation and assembly of quadruplex nucleic acids. Biopolymers.

[56] Dapic V., Abdomerovic V., Marrington R., Peberdy J., Rodger A., Trent J.O., Bates P.J., Dapić V., Abdomerović V., Marrington R. (2003). Biophysical and biological properties of quadruplex oligodeoxyribonucleotides. Nucleic Acids Res..

[57] Petraccone L., Erra E., Esposito V., Randazzo A., Mayol L., Nasti L., Barone G., Giancola C. (2004). Stability and Structure of Telomeric DNA Sequences Forming Quadruplexes Containing Four G-Tetrads with Different Topological Arrangements^†^. Biochemistry.

[58] Feigon J., Koshlap K.M., Smith F.W. (1995). 1H-NMR spectroscopy of DNA triplexes and quadruplexes. Methods Enzymol..

[59] Adrian M., Heddi B., Phan A.T. (2012). NMR spectroscopy of G-quadruplexes. Methods (San Diego, Calif).

[60] Hwang T.L., Shaka A.J. (1995). Water Suppression That Works. Excitation Sculpting Using Arbitrary Wave-Forms and Pulsed-Field Gradients. J. Magn. Reson. Ser. A.

[61] Dalvit C. (1998). Efficient multiple-solvent suppression for the study of the interactions of organic solvents with biomolecules. J. Biomol. NMR.

