# Supplementary material for: New G-Quadruplex-Forming Oligodeoxynucleotides Incorporating a Bifunctional Double-Ended Linker (DEL): Effects of DEL Size and ODNs Orientation on the Topology, Stability, and Molecularity of DEL-G-Quadruplexes
Source: Molecules. 2019 Feb 12;24(3):654. doi: 10.3390/molecules24030654 (PMC6384581; doi:10.3390/molecules24030654)
Supplement: Supplementary file 1 [file molecules-24-00654-s001.pdf]

New G-quadruplex-forming oligodeoxynucleotides incorporating a bifunctional Double-Ended Linker (DEL): effects of DEL size and ODNs orientation on the topology, stability and molecularity of DEL-G-quadruplexes

Maria Marzano [1], Andrea Patrizia Falanga [1], Stefano D'Errico [1], Brunella Pinto [2], Giovanni Nicola Roviello [3], Gennaro Piccialli [1], Giorgia Oliviero[4,*] and Nicola Borbone [1]

[1]Dipartimento di Farmacia, Università degli Studi di Napoli Federico II, via Domenico Montesano, 49 - 80131 Napoli, Italy

[2]Dipartimento di Chimica, Università degli Studi di Milano, via Camillo Golgi, 19 – 20133 Milano, Italy

[3]Istituto di Biostrutture e Bioimmagini, CNR, Via De Amicis, 95, 80145, Napoli, Italy

[4]Dipartimento di Medicina Molecolare e Biotecnologie Mediche, Università degli Studi di Napoli Federico II, via Sergio Pansini, 5 - 80131 Napoli, Italy

***** Correspondence: golivier@unina.it; Tel.: +39-081-679896

SUPPLEMENTARY MATERIAL

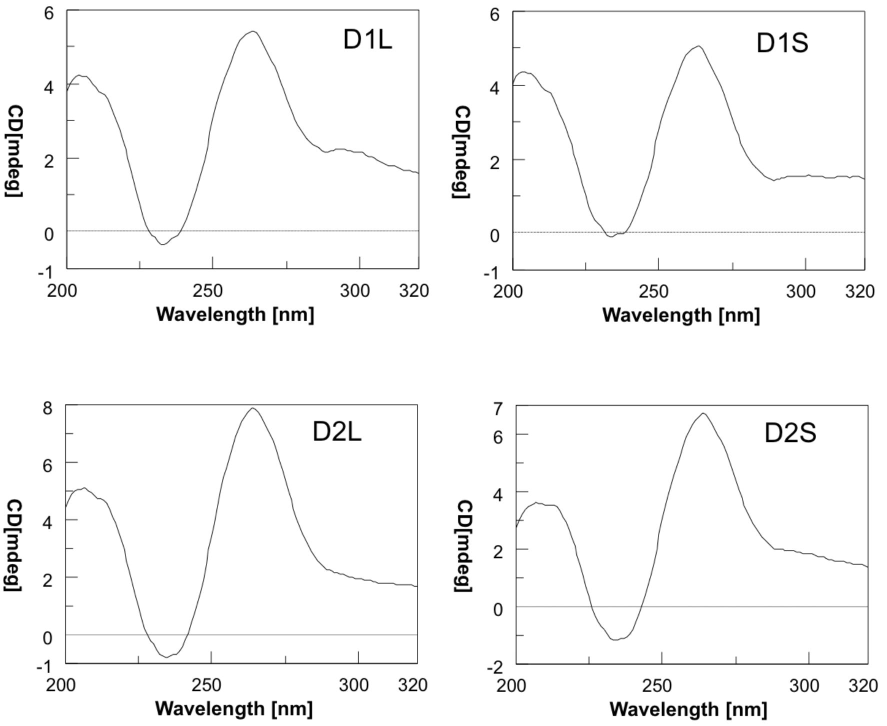

Figure S1: CD spectra recorded at 5 ℃ of **D1L,S** and **D2L,S** annealed in 100 mM Na^{+}-containing buffer.

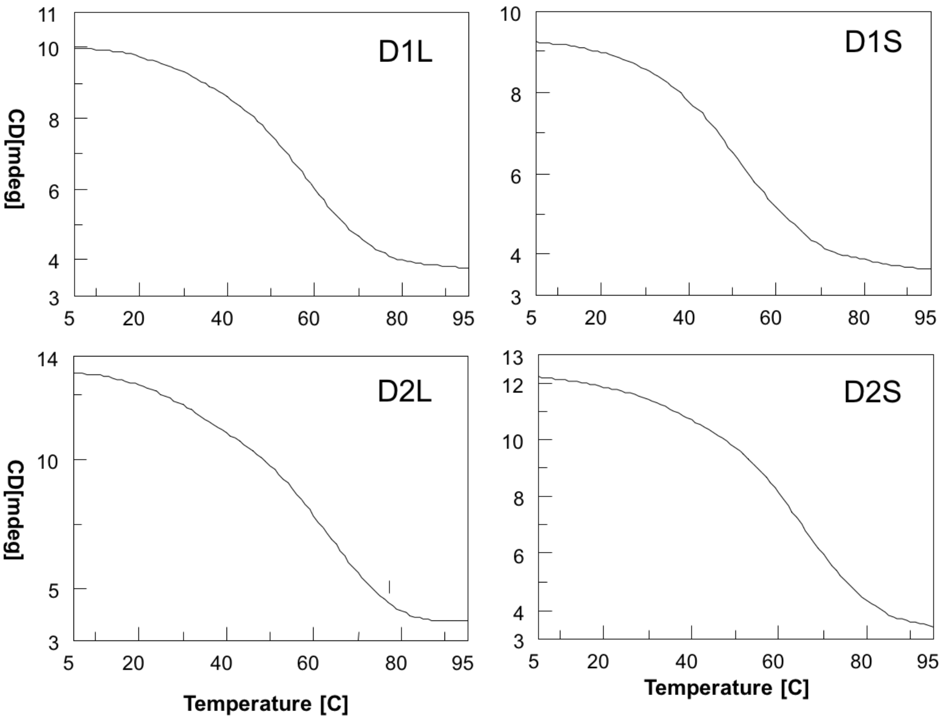

Figure S2: CD value (mdeg) at 263 nm of **D1L,S** and **D2L,S** annealed in 100 mM Na^{+} buffer. Temperature range 5–95 ℃, heating rate 0.5 ℃ min^{-1}.

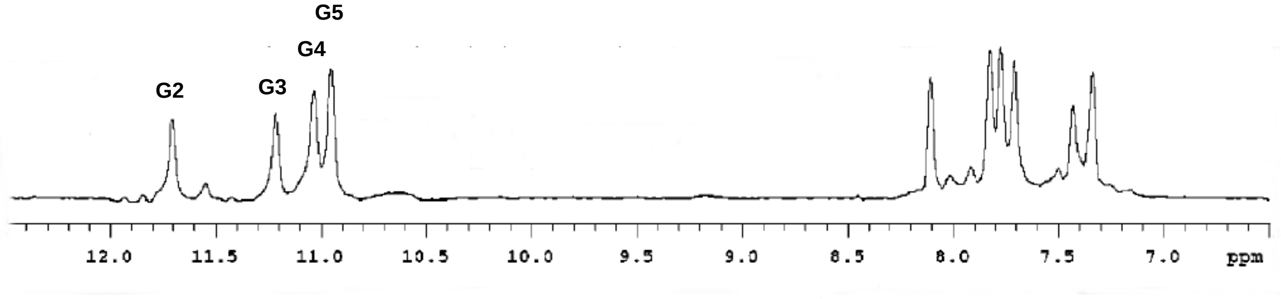

Figure S3. ^{1}H-NMR (500 MHz) spectrum of $[TG4T]_4$ in K^{+} buffer (100 mM KCl and 10 mM K_2HPO_4) recorded in H_2O/D_2O (9:1, v/v). Image taken from ref. 45 in the main text.
